# Experimental Study of Chloride Resistance of Polypropylene Fiber Reinforced Concrete with Fly Ash and Modeling

**DOI:** 10.3390/ma14164417

**Published:** 2021-08-06

**Authors:** Xue-Fei Chen, Chang-Qing Quan, Chu-Jie Jiao

**Affiliations:** School of Civil Engineering, Guangzhou University, Guangzhou 510006, China; chenxuefei@email.szu.edu.cn (X.-F.C.); quanchangqing622@e.gzhu.edu.cn (C.-Q.Q.)

**Keywords:** chloride resistance, fly ash concrete, respond surface model, fuzzy logic model

## Abstract

Herein, the paper reports an experimental investigation lasting one year on the chloride resistance of polypropylene fiber (PF) reinforced concrete with fly ash (FA). Four influential factors at four levels were studied, viz. water to binder ratio (w/b) (0.53, 0.34, 0.29, and 0.25), PF dosage (0%, 0.06%, 0.08%, and 0.1% in volume basis of the total volume of concrete), FA content (0%, 15%, 25%, and 35% in mass substitution ratio of cement) and concentration of NaCl solution (0%, 3%, 5%, and 7%). Dry–wet cyclic immersion and long-term soaking were taken into consideration in addition to the aforementioned factors. A L16(4^4^) orthogonal table was used to sequence influencing factors and to determine the optimal combination. Results showed that 7% NaCl solution caused the highest chloride content in 0–5 mm depth, whilst the w/b ratio of 0.25 curbed the chloride penetration within 10 mm even for concrete subjected to dry–wet cyclic immersion for 360 d. Subsequently, a respond surface model (RSM) basing on polynomials was constructed to visually evaluate the effect of PF dosage and FA content. Results clarified that a cubic model was more precise and PF dosage and FA content turned out to have the positive facilitation to chloride resistance. The positive effect of PF however is not consistent and commensurate for concrete with varied fly ash content. Finally, a fuzzy logic based nonlinear model accommodating all seven influencing factors was verified to be proper and adaptive in predicting chloride content.

## 1. Introduction

The chloride ion is a typical aggressive substance that triggers the corrosion process of steel reinforcement and further causes deterioration, shortening the service life of structures. In the marine environment, those chlorides dissolve in water and ingress to the interior of concrete via a myriad of skeleton and connected pores. To this end, some active mineral additives are blended into concrete to modify the pore structure and connectivity. Among those mineral additives, environmentally friendly and cost-effective industrial byproducts including slag and fly ash are noteworthy. It was reported that the slag powder promoted cement hydration and curbed creep and drying shrinkage, resulting in an enhanced resistance of concrete to chloride penetration [1,2]. The addition of fly ash generated similar effects. A lower level of chloride penetration was verified when 27% fly ash was used [3]; the promoted chloride resistance was attributed to the ink-bottle pores caused by the pore reinforcement effect of fly ash. Chloride ions can be chemically combined by alumina existing in fly ash [4]. A key hydrate, calcium hydroxide, due to the pozzolanic reaction of fly ash can be transformed into C-S-H gel that is more resistant and is benign to curb chloride migration [3,4,5]. The fly ash was also beneficial for workability due to the ball effect [6] and for lowering hydration heat ascribing to the saved cement content [7]. However, Yousuf et al. [8] found that the fracture energy and splitting tensile strength of concrete with fly ash were decreased. It was believed to be caused by the impediment effect generated by aluminum and silicon ions of fly ash on the growing of C-S-H gel and the associated immature pozzolanic reaction [9]. The reduced splitting tensile strength means a limited crack resistance that further marks a higher chloride penetration potentially. To compensate this shortcoming, fibers were used to reinforce concrete with fly ash because fiber reinforcement was an effective countermeasure to lift tensile strength, to confine cracks and to descend the permeability of concrete.

By descending the porosity and ascending the tortuosity, fibers can preserve the impermeable nature of concrete in an uncracked state [10,11,12,13]. Conventional fibers that were used include nylon fiber, steel fiber, carbon fiber, glass fiber, basalt fiber, straw fibers, and so forth [14]. Among those fibers, the polypropylene fiber (PF), attributing to the high abrasion and corrosion resistance and low cost, was a promising reinforcing material using in the alkaline environment where the concrete holds [15,16,17]. The PF was effectively in curbing spalling caused by internal pressure of concrete under high temperature and is beneficial for structural design of elements [18]. Owing to the high tensile strength of PF, the tensile strength and the toughness of concrete can be elevated while the plastic shrinkage and the brittleness are reduced. The three dimensional network system formed by PF in the matrix also function as a skeleton in enduring a higher internal stress and in curbing the expanding and prolongation of cracks, particularly in the early stage [16]. The fiber network effect was also verified by [19]; the fiber orientation was identified as a crucial factor affecting the designing of slab-type elements with plastic fiber reinforced concrete. The above-mentioned reinforcing effects elucidated that PF, when blended into the concrete, controlled the initialization and development of cracks and thus resulted in a better resistance to chloride penetration.

However, the authors of previously published articles seldom studied the integrated effect of fly ash and PF on the chloride penetration of normal and high strength concrete subjected to two environmental factors of dry–wet cyclic immersion and long-term soaking at various corroding concentrations. The novelty of this research is to concurrently conduct in-depth research and model the combined influence of four main influential factors of w/b ratio, fly ash content, PF dosage and soaking concentration of NaCl solution, and one supplementary variable of the environmental condition on the chloride resistance of concrete. In this study, the sequence of significance of different independent variables was identified using range analysis; the optimal condition of each variable was determined. Furthermore, another interesting point was located using respond surface methodology (RSM) to predict the chloride content of PF reinforced concrete with fly ash. By RSM, the effect of fly ash and PF at various levels was visually portrayed.

At last, the diffusion coefficient of chloride ion traditionally is set as a function of corrosive time and penetration depth and is thereby used to predict service life of structures. Nevertheless, the chloride penetration is a multifactorial problem, with influential factors sourcing from the nature of concrete and the exposure environment of the structure. In this study, the former is represented by w/b ratio, the PF dosage, and the fly ash content, whereas the latter is embodied by the concentration of NaCl solution and the distinct corrosive conditions (dry–wet cyclic immersion or long-term soaking). Conventional models of diffusion coefficient subjected to the identified function structure failed to accommodate all aforementioned factors at various levels even though complex mathematical calculations were involved, such as [20,21]. Furthermore, it was proposed that specific design considerations on the fiber types such as the plastic fiber and the steel fiber were essential for the design of fiber reinforced concrete [22]. Consequently, a data-driven model is built herein based on fuzzy logic. The proposed mathematical model is expected to precisely predict the content of chloride ions in specific depths of concrete with varied constituents and in disparate conditions.

## 2. Materials and Methods

### 2.1. Materials

#### 2.1.1. Cement and Fly Ash

The cement is P.O 42.5R, with density of 3.1 g/cm^3^ and Blaine specific surface area of 3250 cm^2^/g. The low lime grade I fly ash was sourced from the Hengyun power station. Its density is 2.2 g/cm^3^ and the specific surface area is 2897 cm^2^/g. The physical properties of cement and chemical composition of cement and fly ash are given in Table 1 and Table 2, respectively.

#### 2.1.2. Aggregates

**Figure 1:**
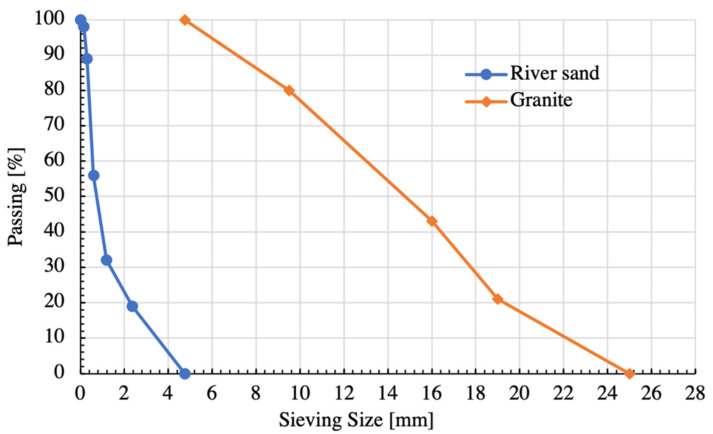
Sieving curve of aggregates.

Dry and clean granite and river sand are used as coarse and fine aggregates, respectively. The size fraction of granite is 5–25 mm, with the apparent density of 2540 kg/m^3^ and the crushing index of 10.1%. The fineness modulus and apparent density of river sand are 2.7 and 2480 kg/m^3^, respectively. The sieving curve of aggregates is depicted by Figure 1.

#### 2.1.3. Polypropylene Fiber (PF) and Water Reducer

The PF was purchased from a local company (Xinhe Co. Ltd., Guangzhou, China), with main properties exhibited in Table 3. The water reducer is CJ-SP8 retarding superplasticizer. It is a brown liquid, with the specific gravity of 1.19 and the water reducing rate of 18%.

### 2.2. Sample Preparation

#### 2.2.1. Mix Proportion and the Design of Orthogonal Experiment

The mix proportion of reference concrete was designed by the unit volume method as per the Chinese national standard “Specification for mix proportion design of ordinary concrete” [23]. Four types of reference concrete from normal strength to high strength were prepared, with component content available in Table 4. Regarding to the four influence factors, viz. w/b, fly ash content, PF dosage, and soaking concentration, a full experiment contained 4^4^ = 256 specific experiments. Instead, the L_16_(4^4^) orthogonal table that covered four factors with four levels was used, with details given in Table 5. Based on Table 4 and Table 5, the mix design of experimental concrete is shown by Table 6. Apart from mixtures in Table 6, some extra batches of concrete were prepared to gather data essential in constructing RSM. On the base of reference concrete with w/b = 0.34, the fly ash was used to replace cement in mass ratios of 0%, 15%, 25%, and 35%, while the PF was in volumetric dosages of 0%, 0.06%, 0.08%, and 0.1% used to reinforce the concrete with fly ash at each replacement level.

#### 2.2.2. Sample Casting and Curing

Raw materials as per Table 6 were put into a SJD60 horizontal concrete mixer and then casted into steel molds with internal dimensions of 100 × 100 × 100 mm. The prepared specimen was covered with a plastic film to prevent moisture dissipation and then demolded after 24 h and next transferred to a standard curing chamber with temperature of 20 ± 3 °C and relative humidity of 95%. The age of standard curing is 28 d.

#### 2.2.3. Soaking by Sodium Chloride (NaCl) Solution

**Figure 2:**
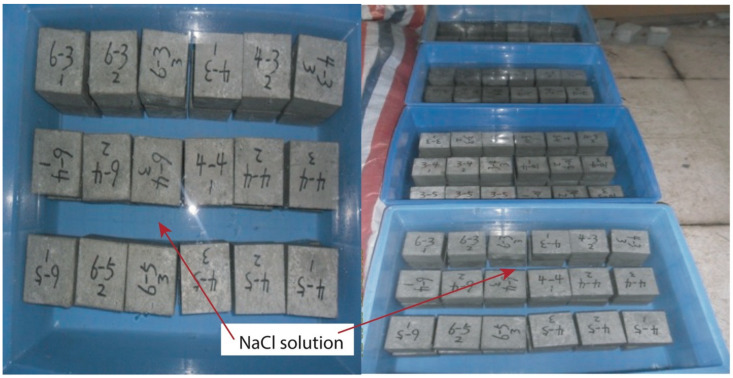
Soaking process of concrete samples.

NaCl solutions at four concentrations as per Table 5 were used to soak and corrode concrete samples. Five sides of samples were sealed by paraffin wax; the bottom side that immersed in the solution was wax-free. The one-sided erosion was expected to accurately reflect the one-dimensional erosion law of chloride ion penetration (see Figure 2). For all concentrations, two environmental conditions (viz. the dry–wet cyclic immersion and long-term soaking) and five erosion periods (viz. 30 d, 60 d, 90 d, 180 d, and 360 d) were applied to samples. A dry–wet cyclic immersion meant to immerse samples in NaCl solution for 3 d (wet condition) and followed by another 3 d drying in the air under room temperature. The cyclic environment was formed by a series of dry–wet cycles. The long-term soaking meant to dip samples in the solution consistently until the testing date. It was in accord with the chloride erosion model of concrete in saturated situation.

### 2.3. Determination of the Content of Chloride Ions

Samples reached designed curing days (30 d, 60 d, 90 d, 180 d, and 360 d) were taken out from the solution and then underwent a series of processes including drilling, slicing, polishing, and chemical titration to determine the level of chloride ion in identified depth (0–2 mm, 2–5 mm, 5–10 mm, 10–15 mm, 15–20 mm, and 20–25 mm), with specific procedures as followings. First, core samples with diameter of 20 mm were drilled from cubic samples by a vertical drilling machine (ZS-50B, Xingguang Company, Jiangyan City). Then, slices were cut from core samples by a low-speed diamond cutting machine (SYJ-160, Kejing Company, Shenyang), with the thickness of slices controlled by a digital adjustable ruler. Three kinds of thickness were used: 2 mm for the first layer, 3 mm for the second layer, and 5 mm for the third and subsequent layers, accurate to 0.01 mm. Next, slices were crushed and ground into powder in a porcelain mortar. Powder passing through the 0.63 mm sieve was oven-dried under 105 ± 5 °C for 2 h and then transferred to a sealed dryer to cool down to room temperature. At length, 2 g powder was weighted by an analytical balance with a sensitivity of 0.0001 g and then placed in a triangular flask. The flask then was filled with 20 mL distilled water, followed by a vibration lasting 1–2 min. The clean sample after 24 h placing was filtered and moved to another triangular flask. After adding two drops of phenolphthalein, the above prepared filtrate presented a color of light red that was then neutralized to colorless with dilute sulfuric acid. 10 drops of potassium chromate were added as an indicator, followed by the titration of silver nitrate solution until the dark red color was achieved. The amount of silver nitrate used was recorded and then the content of chloride ion dissolved in water was calculated by Equation (1) as per [24].

(1) $$P=\frac{C_{AgNO_{3}}\times V_{3}\times 0.03545}{G\times \frac{V_{2}}{V_{1}}}\times 100\%$$

where *P*—content of chloride ion dissolved in water, %; $C_{AgNO_{3}}$—concentration of silver nitrate, mol/L; *G*—weight of samples, g; *V*_1_—amount of water used to immerse samples, ml; *V*_2_—amount of filtrate extracted in each titration, mL; and *V*_3_—amount of silver nitrate consumed by each titration, mL.

### 2.4. Range Analysis Method

**Figure 3:**
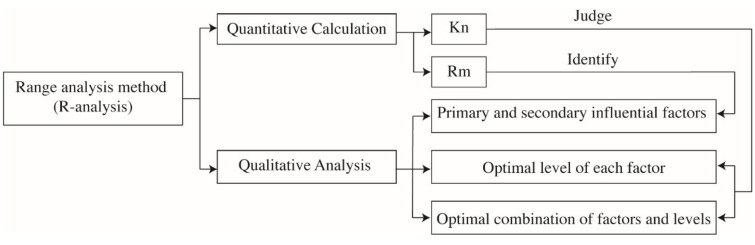
Schematic diagram of the range analysis method.

In collaboration with the orthogonal experimental design, the range analysis is a simple and convenient method to identify the primary and secondary influencing factors, the optimal level of each factor, and the best combination of various factors and levels. The range of each variable is calculated by Equations (2) and (3); the schematic diagram of range analysis methodology is depicted by Figure 3. The range is positively related to the materiality level; the factor with the largest range is the most influential and is the primary factor.

(2) $$K_{n}=\frac{y_{1}+y_{2}+y_{3}+y_{4}}{4}$$

(3) $$R_{m}=\max\left\{Kn\right\}-\min\left\{Kn\right\}$$

where *y_n_* means the test result of the specific variable in the specific level, *n* equals the number of levels; *R_m_* means the range of the specific variable, and *m* equals the number of variables.

### 2.5. Respond Surface Methodology (RSM)

The RSM is a statistical method that establishes a functional relationship between influential factors and the respond by the multiregression. In this study, both the quadratic (Equation (4)) and cubic (Equation (5)) polynomials were used to construct the RSM.

(4) $$z=a+bx+cy+dxy+ex^{2}+fy^{2}$$

(5) $$z=a+bx+cy+dxy+ex^{2}+fy^{2}+gx^{2}y+hxy^{2}+ix^{3}+jy^{3}$$

where *z* is the respond, viz. the content of chloride ion [%]; *x* is the first influential factor, viz. the PF dosage [vol.%]; *y* is the second influential factor, viz. the fly ash content [wt %]; and *a* to *j* are undetermined coefficients.

### 2.6. Adaptive Neuro-Fuzzy Inference System (ANFIS)

**Figure 4:**
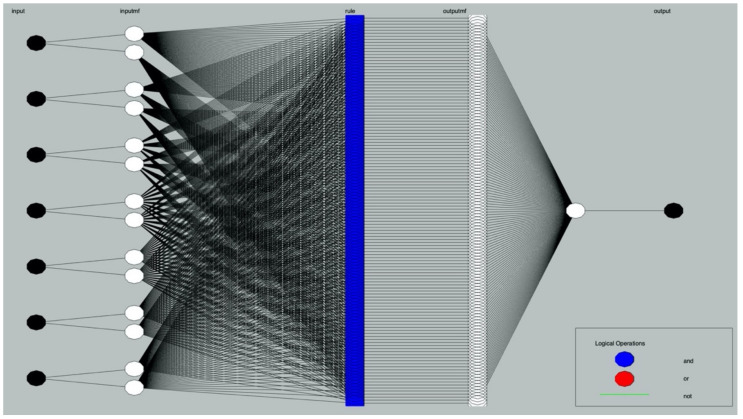
Structure of neuro-fuzzy inference system (ANFIS).

ANFIS is herein used to construct the mathematical model in predicting the content of chloride ions in identified depths of concrete with varied constituents and in different corrosive conditions. A typical ANFIS contains four specific components, viz. the fuzzification of original input data, the establishment of fuzzy rules, the fuzzy reasoning, and the defuzzification of output data (see Figure 4). It is noteworthy that the ANFIS avoids the setting of “hidden layer” that is a tough problem in ANNS. Until now, there is still no universal guiding theory in determining the number of hidden layers and the number of neurons in each hidden layer. It makes researchers spend much time and effort in constantly identifying a proper hidden layer by trial and error; varied settings induce different results with distinct accuracy. The ANFIS based on the fuzzy logic is thereby better in constructing complex nonlinear mapping relations, relative to the conventional artificial neutral network system (ANNS) that functions as a “black box” in data processing. The ANFIS can accommodate multiple input variables and output variables simultaneously, which makes it appropriate in collocation with the respond surface methodology (RSM) that can only visualize two independent factors and one response at a time.

**Figure 5:**
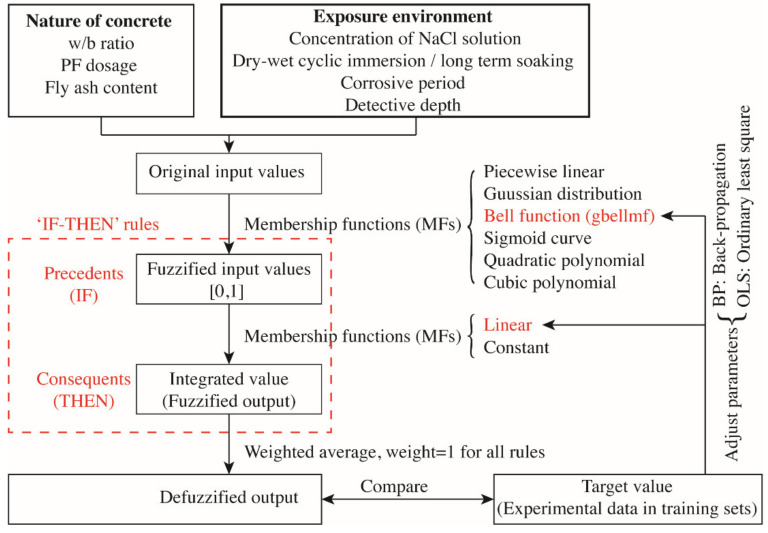
Data processing and algorithms of the neuro-fuzzy inference system (ANFIS).

**Figure 6:**
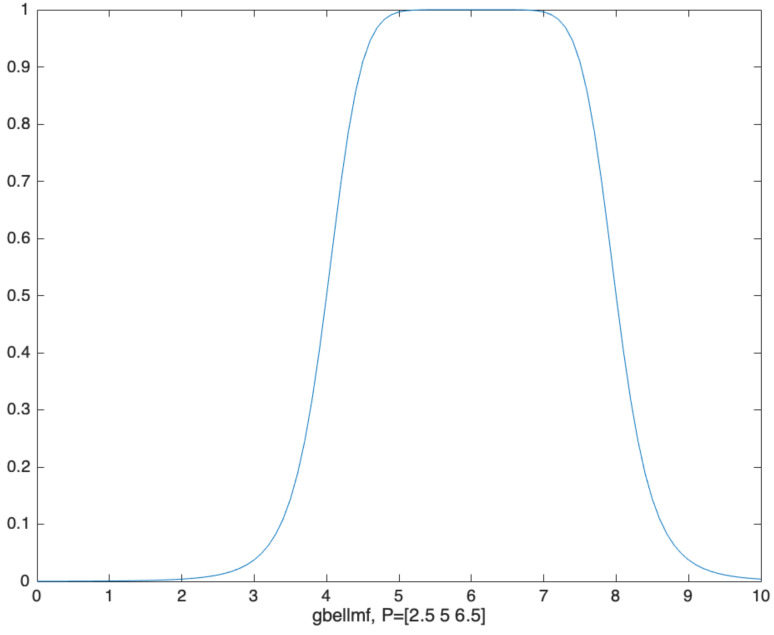
A typical graph of gbellmf (parameters [a, b, and c] respectively equals [2.5, 5, and 6]).

The specific building process of the ANFIS is illustrated by Figure 5 and can be elucidated as followings. First, original data are fuzzified to a value from 0 to 1 by a specific membership function (MF). The commonly used MFs are piecewise linear function, Gaussian distribution function, bell function, sigmoid curve and second-order or third-order polynomial function. Subsequently, fuzzy data are assembled via logical operations of “and”, “or”, and “not” as the “precedents” of the IF-THEN rule. Last, the “precedents” are transformed into the “consequents” through a series of IF-THEN rules. The output data of fuzzy processing are then defuzzified to obtain real output data. Herein, a smooth and zero free bell function (gbellmf) (see Figure 6) with three parameters is used as the MF to map and fuzzify original input values into a range of [0, 1] and the linear MF is used to assemble fuzzified data in each IF-THEN rule. The formulae of gbellmf and linear are respectively presented by Equations (6) and (7) and a typical graph of gbellmf is portrayed by Figure 5. By a hybrid algorithm integrating backpropagation and ordinary least square, the defuzzified output data are compared with the target data sourced from the experimental study and other published articles [4,20,21,25,26]. A total of 400 data sets were collected, with 360 groups setting as the training set and the other 40 groups as testing sets.

(6) $$gbellmf\left(x,\;\left[a,\;b,\;c\right]\right)=\frac{1}{{\left(1+abs\left(\frac{x-c}{a}\right)\right)}^{2\times b}}$$

where *x* is the independent variable and [*a*, *b*, *c*] is the matrix of parameters.

(7) $$linear=ax_{1}+bx_{2}+cx_{3}+dx_{4}+ex_{5}+fx_{6}+gx_{7}+h\;$$

where *x_i_* (I = 1 to 7) means the input value of each variable and [*a, b, c, d, e, f, g, and h*] are the parameters.

### 2.7. Performance Evaluation Criteria

As suggested by [27], three statistical index, viz. the root mean square error (RMSE), correlation coefficient (*R*^2^), and the mean absolute error (MAE) were used as criteria in evaluating the performance of the established mathematical model, with specific mathematical expressions available in Equations (8)–(10). Statistically, a smaller RSME or MAE (0 is the best) and a larger *R*-squared value (1 is the best) means a better prediction performance of the model.

(8) $$RMSE=\sqrt{\frac{\sum_{i=1}^{n}{\left(\bar{x’}-x^{\prime }\right)}^{2}}{n}}$$

(9) $$MAE=\frac{\sum_{i=1}^{n}\left|\bar{x’}-x^{\prime }\right|}{n}$$

(10) $$R^{2}=1-\frac{\sum_{i=1}^{n}{\left(\bar{x’}-x^{\prime }\right)}^{2}}{\sum_{i=1}^{n}{\left(\bar{x}-x\right)}^{2}}$$

where, *x*, $\bar{x}$, and $x^{\prime }$ indicate to the experimental value, the average of values, and the predicted value, respectively.

## 3. Results and Discussion

### 3.1. Effect of Corrosive Environment (Dry–Wet Cycling vs. Long-Term Soaking) on Chloride Resistance

**Figure 7:**
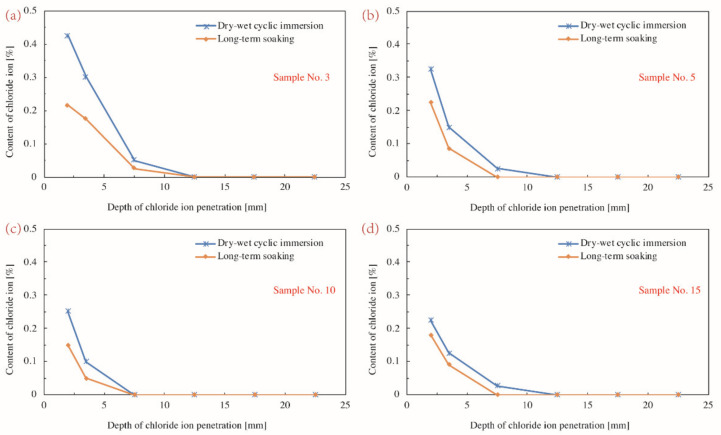
Depth of chloride ion penetration and the content of chloride ion in various depths of concrete samples corroded for 30 d: (**a**) Sample No.3; (**b**) Sample No.5; (**c**) Sample No. 10; (**d**) Sample No. 15.

Figure 7 illustrates the penetration depth and content of chloride ions in concrete samples corroded for 30 d under different conditions. It is noted that the dry–wet cyclic immersion triggered a more severe corrosion relative to the long-term soaking condition, with other factors fixed. On one side, the corrosion depth under the long-term soaking situation scarcely exceeded 10 mm; the depth of concrete suffered dry-wet cycling even approached to 15 mm (see Figure 7d). On the other side, the content of chloride ion of concrete under dry–wet cyclic environment in 2 mm and 3.5 mm depth were 0.43% and 0.3%, which were almost twice as much as the content (0.22% and 0.18%) of samples in corresponding depth in the long-term soaking condition (see Figure 7a). This varied level of corrosion was believed to be caused by the disparate mass transfer process of chloride ion under different circumstances.

The dry–wet cyclic environment accelerated the corrosion because the concentration gradient, the temperature gradient, and the humidity gradient concurrently coexisted in the interior of concrete. A severe corrosion was also observed on galvanized steel when a wet–dry cyclic environment applied to it [28]. Chloride ions under the complex coupling effect of diffusion and convection in a relatively higher speed penetrated from the outside into the inside. Water in pores flowed and formed seepage, while chloride ions moved in pace with the pore water simultaneously, forming convection. The mass transfer process of chloride ions in this unsaturated porous media was thereby controlled both by the diffusion and the convection. When concrete that undergone a drying period was immersed into the NaCl solution again, the absolutely dry or partially dry part would absorb the water as well as the chloride ions until the saturated status attributing to the absorptive capillary action was reached. If the concrete was deeply dried, the subsequent wet cycle could bring chloride ions into a greater depth and accelerated the invasion of chloride ion. When going through another dry period, chloride ions continued to invade into the concrete due to the diffusion effect. This kind of penetration would not stop until the saturation status at a certain depth reached. Together with the chloride ions accumulation caused by the water evaporation, the measured water-soluble chloride concentration was basically higher than that of the same kind of concrete in the long-term immersion test group.

On the contrary, the long-term soaking was in accord with the chloride erosion model of concrete in the state of complete saturation because pores of concrete were always saturated by pore water. There was only concentration gradient between the surface and the interior of the concrete in the environment where chloride ions were completely saturated. In this instance, the mass transfer process of chloride ions was dominated by diffusion, while ions diffused from the high concentration to the low concentration to reach balance, viz. from the surface to the interior. It was reported that chloride ions when diffused into the cement paste resulted in the Friedel’s salt (3CaO. A1_2_O_3_. CaCl2. 10H_2_O) that retarded the process of further diffusion and decreased aqueous ratios [29]. C-S-H gel generated by hydration of cement adsorbed chloride ions in three forms: on the surface of the gel, in the interlayer of gel, or tightly bonded with the gel. The transportation of chloride ions in concrete thereby was accompanied by the combination and adsorption of chloride ions and the diffusion speed of chloride ions in concrete was affected correspondingly. The above makes the driving force caused only by diffusion much weaker than the impetus sourced from the combined diffusion and convection, which explained the varied penetration depths and content of chloride ion in concrete under different corrosive environment.

Furthermore, the foresaid regularity seems to be appropriated to all samples regardless of the specific composition, as is portrayed by subgraphs Figure 9a–d. It is because the essence of the mass transfer process of chloride ion is only affected by the environment condition. However, the specific constituent component and associated dosage still affect the degree of deterioration to a certain extent. For example, when the w/b ratio lowered from 0.53 to 0.34, the content of chloride ion under the dry–wet cycling reduced by 23.5% at the detecting depth of 2 mm and by around 50% at the depth of 3.5 mm. A similar phenomenon was also observed for the concrete suffering long-term soaking. The ion content of long-term soaking samples only retained half at the 3.5 mm depth. The 50% slip was attributed to a denser structure induced by the low w/b ratio. A detailed discussion on the effect of other factors such as the fly ash content and the PF dosage on the content of chloride ion is available in following sections.

### 3.2. Range Analysis of the Effect of Various Influencing Factors on the Chloride Ion Content

**Figure 8:**
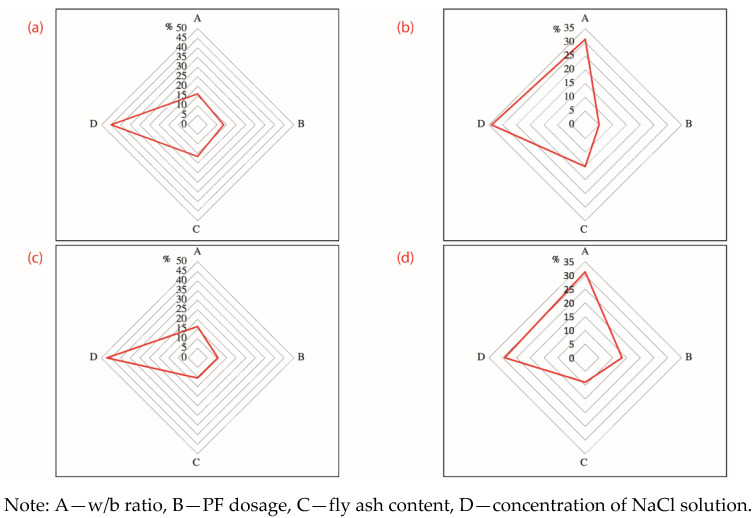
Range analysis of the content of chloride ion of concrete eroded for 30 d (**a**) 0–2 mm concrete samples suffering long-term soaking; (**b**) 2–5 mm concrete samples suffering long-term soaking; (**c**) 0–2 mm concrete samples suffering dry-wet cycling; (**d**) 2–5 mm concrete samples suffering dry–wet cycling.

Figure 8 visualizes the range analysis of factors that affect the chloride ion content of concrete samples long-term soaked or dry–wet cyclic immersion for 30 d in the NaCl solution, with two observation depths of 0–2 mm and 2–5 mm. The detective depth was set 0–2 mm and 2–5 mm because the content of chloride ion is slight and not detectable when the test depth further deepens into 5–10 mm and deeper. Range analysis as a qualitative analytical tool is used to identify the primary and the secondary influencing factors, as highlighted by Figure 5. In this instance, a mathematical transformation as per Equation (11) was applied to original values of ranges. The transformed ranges are easy to be compared with each other and are more suitable to be presented by radar maps.

(11) $$R_{i,new}=\frac{R_{i}}{R_{A}+R_{B}+R_{C}+R_{D}}\times 100\%$$

where *R_i_* is the original value of range of influential factor *i* and *i* can be *A*, *B*, *C*, and *D*; *R_i,new_* is the transformed value of range of influential factor *i*; and *i* can be *A*, *B*, *C* and *D*.

It is found in Figure 10a that factor D (concentration of NaCl solution) is the primary influencing factor, followed by C (fly ash content), A (w/b ratio) and B (PF dosage). Factor D has a highly significant effect on the content of water-soluble chloride ion in concrete, while factor A, factor B, and factor C have almost equal and insignificant clout. Regarding factor D specifically, D4 (7%) > D3 (5%) > D2 (3%) > D1 (0%), that is, the higher the concentration of NaCl solution, the higher the content of water-soluble chloride ion in concrete. It is because a higher NaCl concentration indicates to a corresponding higher level of free chloride ions and a higher concentration gradient. In the long-term soaking condition, the mass transfer is diffusion dominated. A higher initial concentration accelerates the diffusion since diffusion flux is positively proportional to the gradient of concentration basing on the Fick’s second law [30,31,32].

**Figure 9:**
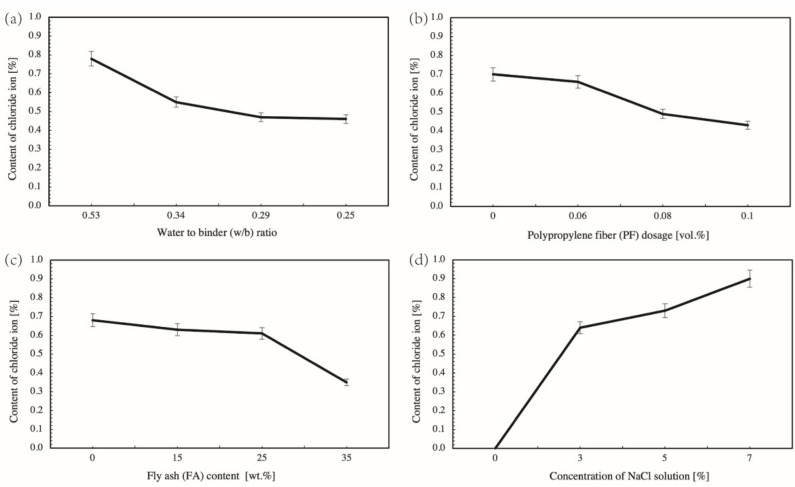
Influence of various factors on the content of chloride ions in 0–2 mm depth of concrete samples suffered dry–wet cyclic immersion for 30 d. (**a**) Water to binder (w/b) ratio, (**b**) Polypropylene fiber (PF) dosage [vol.%], (**c**) Fly ash (FA) content [wt.%], (**d**) Concentration NaCl solution [%].

**Figure 10:**
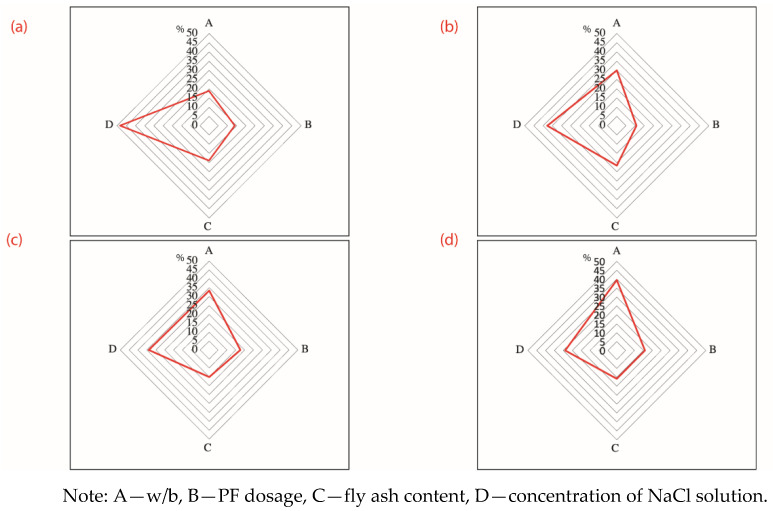
Range analysis of influential factors on the content of chloride ion in various depths of concrete samples eroded for 360 d under the wet–dry cyclic immersion condition: (**a**) 0–2 mm; (**b**) 2–5 mm; (**c**) 5-–10 mm; (**d**) 10–15 mm.

However, with others fixed, the aforementioned finding varies when the observation depth deepens from 0–2 mm into 2–5 mm. The new sequence of importance of factors turns into D (concentration of NaCl solution) > A (w/b ratio) > C (fly ash content) > B (PF dosage) (see Figure 10b). Factor D still has the most significant effect on the water-soluble chloride ion content of concrete and Factor B as before has no evident effect on experimental results. However, the importance of Factor A and Factor C strengthens, pushing the w/b ratio and the fly ash content become the second and the third primary influencing factor, respectively. It is notable that Factor A is comparable to the concentration of NaCl solution and is even more prominent regarding to samples in the dry–wet cyclic immersion condition (see Figure 9d). It is because the w/b ratio is a key factor in determining the compactness of concrete; a lower w/b ratio means a higher densification of concrete and a harder accessibility of chloride ions. This finding is consistent to a previous study that observed a reduced chloride diffusion depth and a declined chloride ion diffusion coefficient for cement pastes with a lower w/c ratio [33]. The C-S-H gel generated by the hydration adsorbed chloride ions, accounting for a great retardation to the diffusion of chloride ions [29,34]. The rising influence of fly ash sources from the microaggregate filling and pozzolanic effects. Hu et al. [35] once used supplementary cementitious materials including slags, fly ash, and silica fume in mitigating the chloride penetration and reported that the fly ash densified the pore structure and promoted the chloride resistance. Fly ash was proved to improve the performance of cementitious materials due to the physical effect of filling and the chemical effect of pozzolanic reaction. The aluminate phase of fly ash reacted with calcium hydroxide existing in matrix and generated extra C-S-H gel. The gel on one hand refines the microstructure; on the other hand, it functions as a “sink” to accommodate free chloride ions [4,5,21]. The compacted structure and the retarding effect jointly promote the resistance to chloride ion penetration.

Figure 9 shows the influence of various factors on the content of chloride ions in 0–2 mm depth of concrete samples suffered dry–wet cyclic immersion for 30 d. It is observed that 0.08% PF dosage yields a 29.8% reduction of chloride content. It is because soft fibers such as PF fiber and the basalt fiber can reduce the porosity and decreases the chloride penetration [13]. The PF fiber was reported to dense the internal structure of cementitious materials by decreasing the whole porosity and the volume of large pores [36]. However, a further increase of PF does not evidently lead to a lower chloride again. It is because a higher dosage of fibers potentially leads to the twining and aggregation, which causes more interfaces with the surrounding matrix and forms extra voids. The marginal diminishing effect of higher content of fibers was also reported by [13,36]. Furthermore, it was reported that plastic fibers triggered a flexural creep coefficient that is more than double than that of steel fiber [37]. The associated postcracking behavior of PF reinforced concrete is detrimental to curb the penetration and diffusion of chloride ions. In addition, a previous study verified that polypropylene based macrofibers exacerbated the chloride ion penetration in a higher volume dosage [1]. The optimal level of PF fiber thereby is identified as 0.08% in the volumetric basis of the concrete. It results in the optimal level combination of [w/b ratio, PF dosage, fly ash content] as [0.25, 0.08%, 35%].

Figure 10 shows the ranges of various influential factors. The pattern overall is similar to that of Figure 10 because the nature of concrete is not essentially changed. However, as the corrosive period prolongs, chloride ions penetration deepens and the content of chloride ions in depth 5–10 mm and 10–15 mm is depicted by Figure 10c,d. It is interesting to note that, as the detective depth deepens, the factor of w/b ratio is more and more influential. Due to the limited water used, the core of concrete with low w/b is dense and hard and thus restrains the penetration of external chloride ions. This observation is consistent to a previous study that stated that a lower w/c ratio resulted in a declined chloride ion penetration [3].

**Figure 11:**
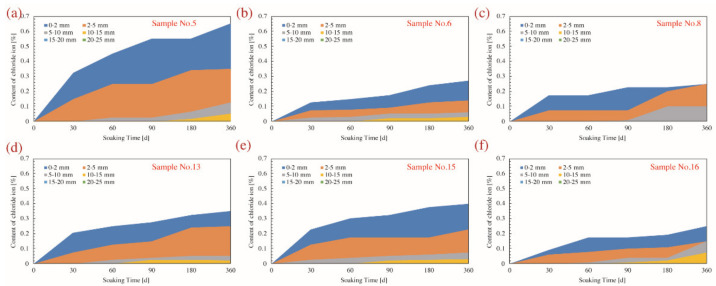
Depth of chloride ion penetration and the content of chloride ion of concrete samples corroded for various days under the dry–wet cyclic immersion environment. (**a**) Sample No.5; (**b**) Sample No.6; (**c**) Sample No.8; (**d**) Sample No.13; (**e**) Sample No.15; (**f**) Sample No.16.

**Figure 12:**
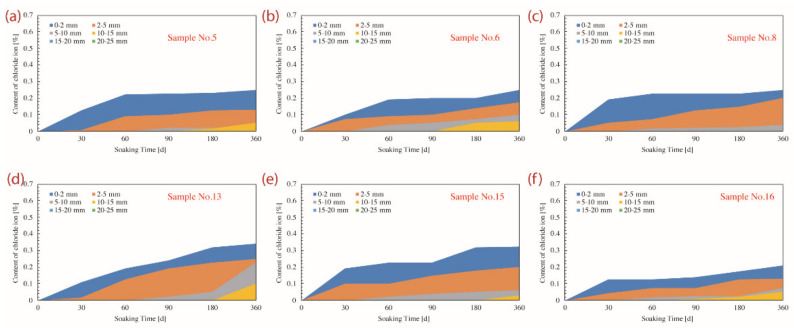
Depth of chloride ion penetration and the content of chloride ion of concrete samples corroded for various days under the long-term soaking environment. (**a**) Sample No.5; (**b**) Sample No.6; (**c**) Sample No.8; (**d**) Sample No.13; (**e**) Sample No.15; (**f**) Sample No.16.

The profiles of penetration depth and exact content of chloride ions are available in Figure 11 and Figure 12. It is noted that the chloride penetration gradually deepened as the corrosive period prolonged, while the penetration depth of all samples was strictly limited within 20 mm even referring to the dry–wet cyclic immersion for 360 d.

### 3.3. RSM

**Figure 13:**
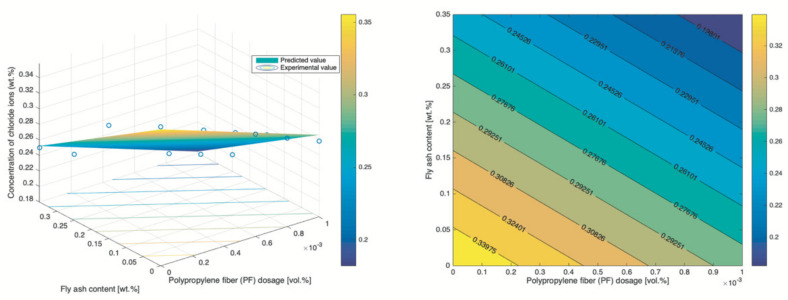
Respond surface and contour map of the quadratic model.

**Figure 14:**
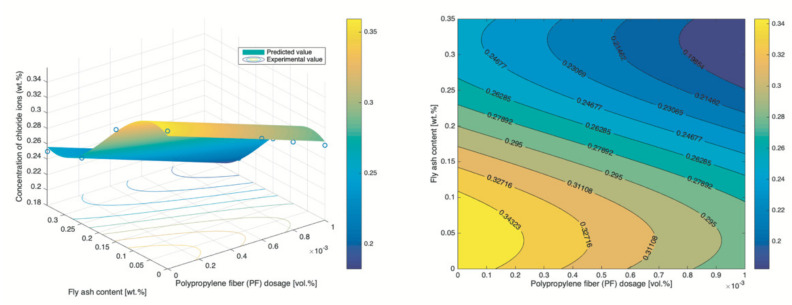
Respond surface and contour map of the cubic model.

**Figure 15:**
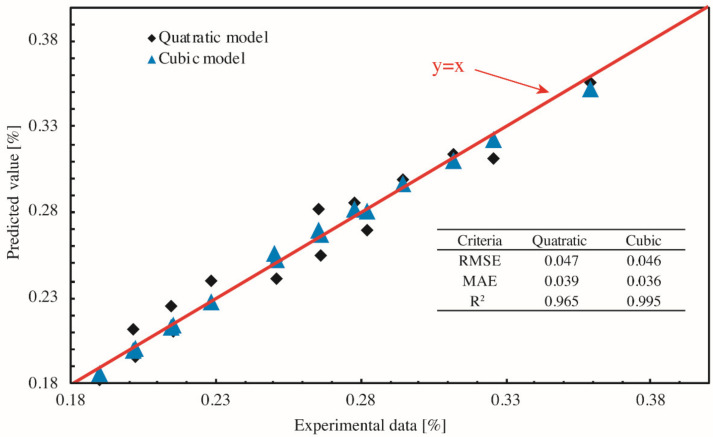
Performance of prediction.

The RSM is used to model the content of chloride ions of concrete prepared by various PF dosages and fly ash content, with w/b ratio and concentration of NaCl solution respectively fixed to 0.34% and 5%. Samples were corroded for 30 d under the dry–wet cyclic immersion condition. The analysis of variance (ANOVA) was in advance used to identify terms that significantly correlated to the respond (chloride content), with Table 7 and Table 8 respectively showing the result of quadratic model and the cubic model. Once significant terms were determined, the corresponding respond surfaces and contour maps could be pictured, with Figure 13 for quadratic model and Figure 14 for cubic model. By the performance evaluation highlighted in Figure 15, the cubic model is better even though both models exhibit a high level of statistical reliability and thus is used for further analysis. Besides the mathematical analysis, the cubic model is better because PF was a prominent factor affecting the content of chloride ion and PF refined the pores and triggered more CSH gel, leading to a denser microstructure.

The quadratic polynomial is shown by Equation (12).

(12) z=0.36−69.99x−0.29y

The cubic polynomial is shown by Equation (13).

(13) $$z=0.35-69.99x+0.36y-5.18y^{2}+9.58y^{3}$$

By Figure 14, both the PF and fly ash can boost the chloride resistance, which echoes the finding in the previous section. However, the effect of PF is not consistent and is affected by the fly ash content. Most notably, an interesting observation is that a lower content of fly ash coupled with a higher dosage of PF result in a respond that is comparable to that of the combination with higher content of fly ash plus lower dosage of PF. For example, ~5% fly ash + ~0.09% PF leads to 0.295% chloride content and ~20% fly ash + ~0.01% PF causes the same level of corrosion. It means that the concrete with low fly ash requires a higher dosage of PF to reach the similar level of chloride ion content that is achieved by the concrete with high content of fly ash. It also reveals that the PF is more effective in lifting the chloride resistance of concrete with lower content of fly ash. Considering the fact that fly ash is a byproduct that has low cost, a higher content of fly ash is cost-effective.

### 3.4. ANFIS

**Figure 16:**
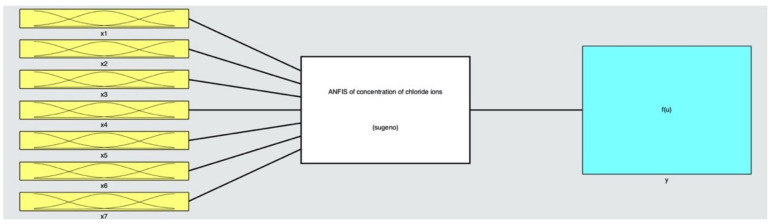
Schematic diagrams of neuro-fuzzy inference system (ANFIS).

Regarding to the problem of chloride resistance, seven influential factors and one dependent variable are taken into consideration in the ANFIS, with details in Table 9 and schematic diagrams in Figure 16.

**Figure 17:**
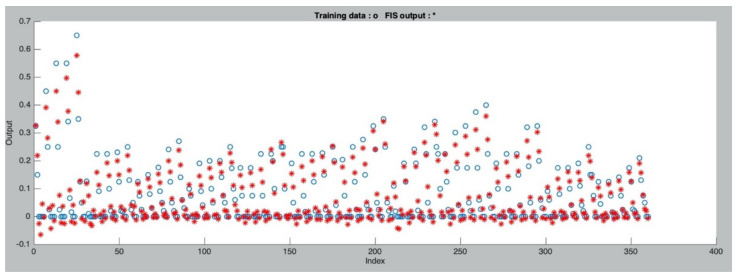
Performance of training data.

With the identified input MF, the matrix of number of MFs is set as [2 2 2 2 2 2 2]. It means each input variable has two specific MFs used for fuzzification. A total of 400 data sets sourced from the experimental study and other published articles [4,20,21,25,26] were used to construct and examine the ANFIS. Among those selected data sets, 360 groups were used as the training set, with the performance of training illustrated by Figure 17. It is noted that the most of output values of ANFIS are approaching to the experimental values, while some outputs are even highly overlapped over the actual values, indicating a good training performance of the established ANFIS.

The basic information of trained ANFIS is as followings. Parameters of gbellmf and linear are given by Table 10 and Table A1 (Appendix A), respectively.

- Number of nodes: 294
- Number of linear parameters: 1024
- Number of nonlinear parameters: 42
- Total number of parameters: 1066
- Number of training data pairs: 360
- Number of checking data pairs: 120
- Number of fuzzy rules: 128

**Figure 18:**
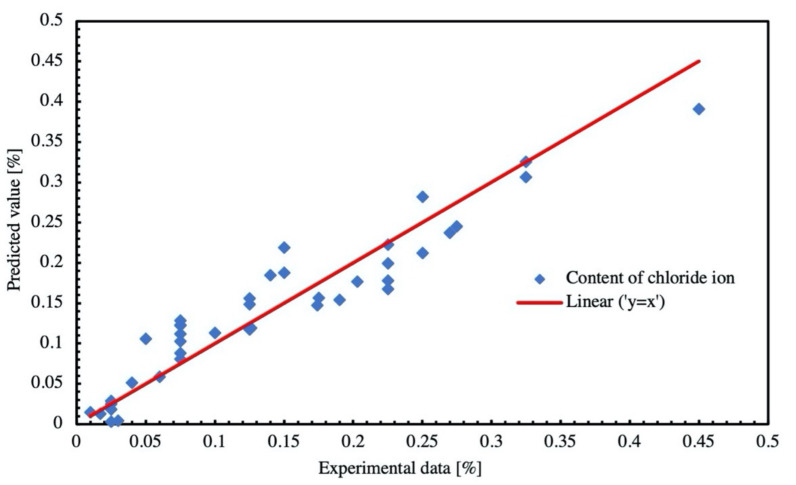
Performance of testing of ANFIS model.

The other 40 sets were used in testing, and the performance of testing is depicted by Figure 18. The *R*-squared value is calculated as 0.89. The value is higher than 0.7 that is usually set as a benchmark in engineering [38], which means the established ANFIS is feasible to be used in predicting chloride content of concrete with high accuracy.

## 4. Conclusions

The paper reported an experimental study lasting one-year on chloride penetration of PF reinforced concrete with fly ash and constructed some mathematical models basing on the RSM and fuzzy logic system. For a comprehensive understanding of the penetration and diffusion of chloride ions in concrete, three interior factors—w/b ratio, PF dosage, and FA content—and two external environment factors—NaCl concentration and curing condition—were selected. The concrete with a w/b ratio of 0.25 restrains the chloride penetration within 10 mm even considering a 360 d dry–wet cyclic immersion, attributing to the dense structure of concrete and the sinking effect of CSH gel on chloride ions. Relying on the filling effect and the pozzolanic reaction, FA content is positive related to the chloride resistance. However, the effect of PF is more prominent at the low dosage because PF potentially triggers postcracking due to a higher flexural creep coefficient. The concentration of NaCl solution is the most significant influential factor for all specimens because a higher concentration means a corresponding higher content of free chloride ions that potentially penetrate into the concrete via the mass transfer process. Relative to the long-term soaking situation, the dry–wet cyclic immersion causes a more severe influence on concrete because chloride ions in the surrounding solution can invade into the interior through the coupled action of the diffusion by the concentration gradient and the convection sourced from the humidity gradient. When it comes to the models, the cubic-polynomial-based RSM proves to be a better model in visualizing the effect of PF dosage and fly ash content on chloride content. The increase of either PF or fly ash shifts chloride content toward a lower level, whereas the impetus of PF is more prominent for concrete with lower content fly ash. The ANFIS that contains 128 IF-THEN rules is instead verified to be a precise model in modeling chloride content that is concurrently affected by seven influential factors.

## Figures and Tables

**Table 1:** Physical properties of cement.

Water Requirement of Normal Consistency.		26.50%	
Setting Time (min)		Initial	236.0
		Final	305.0
Stability		Qualified	
Strength	Curing days (d)	3.0	28.0
	Flexural strength (MPa)	5.2	7.4
	Compressive strength (MPa)	27.2	47.5

**Table 2:** Chemical composition of cement and fly ash.

Oxide	SiO_2_	Al_2_O_3_	Fe_2_O_3_	CaO	MgO	SO_3_	K_2_O	Na_2_O	LOL
Cement	19.71	6.28	3.73	62.91	2.54	2.72	0.90	0.25	0.96
Fly ash	52.50	27.25	5.34	7.17	2.54	0.20	0.97	0.49	3.54

**Table 3:** Physical properties of polypropylene fiber (PF).

Notation	Tensile Strength/MPa	Elastic Modulus	Specific Gravity /g·cm^−3^	Melting Point	Ignition Point	Length	Diameter
		/MPa		/°C	/°C	/mm	/µm
PF	>270	>3500	0.91	160	580	19	30–40

**Table 4:** Mix proportion of reference concrete.

w/b	Water	Cement	Sand	Granite	Sand Ratio	Water Reducer
	Kg/m^3^				%	%
0.53	180	340	745	1090	0.41	2.00
0.34	155	455	660	1095	0.38	2.30
0.29	145	502	600	1080	0.36	2.60
0.25	140	555	612	1137	0.35	2.80

**Table 5:** Orthogonal layout of four factors with four levels.

Exp. No.	A	B	C	D
1	1 (0.53)	1 (0)	1 (0)	1 (0)
2	1	2 (0.06%)	2 (15%)	2 (3%)
3	1	3 (0.08%)	3 (25%)	3 (5%)
4	1	4 (0.10%)	4 (35%)	4 (7%)
5	2 (0.34)	1	2	3
6	2	2	1	4
7	2	3	4	1
8	2	4	3	2
9	3 (0.29)	1	3	4
10	3	2	4	3
11	3	3	1	2
12	3	4	2	1
13	4 (0.25)	1	4	2
14	4	2	3	1
15	4	3	2	4
16	4	4	1	3

**Note:** A is the water to binder ratio (w/b) of reference concrete; B is the volume ratio of polypropylene fiber (PF) in the concrete; C is the weight replacement ratio of fly ash on cement; D is the concentration of solution that is used to soak samples.

**Table 6:** Mix proportion of experimental concrete (kg/m^3^).

Exp. No.	w/b	Water	Cement	Fly Ash	Sand	Granite	PF	Water Reducer
1	0.53	180	340	-	745	1090	-	6.80
2	0.53	180	289	51	745	1090	0.68	7.41
3	0.53	180	255	85	745	1090	0.91	7.82
4	0.53	180	221	119	745	1090	1.14	8.23
5	0.34	155	387	68	660	1095	0.69	10.47
6	0.34	155	455	-	660	1095	-	11.41
7	0.34	155	296	159	660	1095	1.14	12.03
8	0.34	155	341	114	660	1095	0.91	12.66
9	0.29	145	377	126	600	1080	0.91	12.55
10	0.29	145	326	176	600	1080	1.14	13.68
11	0.29	145	502	-	600	1080	-	14.43
12	0.29	145	427	75	600	1080	0.68	15.19
13	0.25	140	361	194	612	1137	1.14	16.18
14	0.25	140	416	139	612	1137	0.91	17.64
15	0.25	140	472	83	612	1137	0.69	18.61
16	0.25	140	555	-	612	1137	-	19.58

**Table 7:** Results of analysis of variance (ANOVA) and regression with quadratic polynomial.

Terms		ANOVA		Regression		
		Prob. > F		Coefficient	[95% Conf. Interval]	
Linear	*x*	0.02	*	−69.99	−85.02	−54.96
	*y*	0.00	*	−0.29	−0.34	−0.25
Quadratic	*xy*	0.29	-	-	-	-
	*x^2^*	0.86	-	-	-	-
	*y^2^*	0.80	-	-	-	-
Constant	-	-	-	0.36	0.34	0.37

Note: * means the term is significant (*p*-value < 0.05), *x* means PF dosage, *y* means fly ash content.

**Table 8:** Results of analysis of variance (ANOVA) and regression with cubic polynomial.

Terms		ANOVA		Regression		
		Prob. > F		Coefficient	[95% Conf. Interval]	
Linear terms	*x*	0.00	*	−69.99	−75.69	−64.29
	*y*	0.00	*	0.36	0.20	0.53
Quadratic terms	*xy*	0.09	-	-	-	-
	*x^2^*	0.45	-	-	-	-
	*y^2^*	0.00	*	−5.18	−6.44	−3.93
Cubic terms	*x^2^y*	0.84	-	-	-	−
	*xy^2^*	0.93	-	-	-	-
	*x^3^*	0.53	-	-	-	-
	*y^3^*	0.00	*	9.58	7.26	11.91
Constant	-	-	-	0.35	0.35	0.36

Note: * means the term is significant (*p*-value < 0.05), *x* means PF dosage, *y* means fly ash content.

**Table 9:** Variables and designations in the adaptive neuro-fuzzy inference system (ANFIS).

Variable	Unit	Designation	Minimum	Maximum
w/b ratio	N/A	x1	0.25	0.53
PF dosage	vol.%	x2	0.00	0.10
FA content	wt.%	x3	0.00	35.00
Concentration of NaCl solution	%	x4	0.00	7.00
Corrosive condition	N/A	x5	0.00	1.00
Detective depth	mm	x6	2.00	22.50
Corrosive period	d	x7	30.00	360.00
Concentration of chloride ions	wt.%	y	0.00	0.65

**Note**: The corrosive condition is a dummy variable, with “1” and “0” meaning the “dry–wet cyclic immersion” and the “long-term soaking”, respectively.

**Table 10:** Membership functions and parameters—gbellmf.

Variable	Range	gbellmf	Notation of mf in “Rules”	Parameters of mf		
				a	b	c
x1	[0.25 0.53]	mf1	1	0.045	2.000	0.250
	[0.25 0.53]	mf2	2	0.045	2.000	0.340
x2	[0 0.001]	mf1	1	0.005	2.000	−0.001
	[0 0.001]	mf2	2	−0.002	2.000	0.003
x3	[0 0.35]	mf1	1	0.175	2.000	0.000
	[0 0.35]	mf2	2	0.175	2.000	0.350
x4	[0.03 0.07]	mf1	1	0.020	2.000	0.030
	[0.03 0.07]	mf2	2	0.020	2.000	0.070
x5	[0 1]	mf1	1	0.500	2.000	0.000
	[0 1]	mf2	2	0.500	2.000	1.000
x6	[2 22.5]	mf1	1	10.250	1.980	1.993
	[2 22.5]	mf2	2	10.300	2.010	22.500
x7	[30 360]	mf1	1	165.000	2.000	30.000
	[30 360]	mf2	2	165.000	2.000	360.000

## Data Availability

Data sharing not available.

## Appendix A

**Table A1:** Membership functions and parameters—Linear.

Rules No.	Type of Input Membership Functions							Parameters of Output Membership Functions (Linear)							
								a	b	c	d	e	f	g	h
	x1	x2	x3	x4	x5	x6	x7	x1	x2	x3	x4	x5	x6	x7	Constant
1	1	1	1	1	1	1	1	0.0051	0.0000	0.0063	0.0011	−0.0016	−0.0023	−0.0001	0.0180
2	1	1	1	1	1	1	2	−0.0210	0.0000	−0.0121	−0.0022	−0.0239	0.0126	0.0002	−0.0696
3	1	1	1	1	1	2	1	0.0136	0.0000	0.0142	0.0023	0.0014	−0.0020	0.0001	0.0459
4	1	1	1	1	1	2	2	−0.0054	−0.0001	0.0163	−0.0015	−0.0109	0.0003	0.0000	−0.0433
5	1	1	1	1	2	1	1	−0.0040	−0.0001	0.0091	−0.0011	−0.0277	−0.0014	0.0013	−0.0265
6	1	1	1	1	2	1	2	−0.1055	−0.0004	0.0005	−0.0204	−0.4070	0.0520	0.0022	−0.4097
7	1	1	1	1	2	2	1	0.0114	0.0000	0.0140	0.0015	0.0246	−0.0014	−0.0003	0.0272
8	1	1	1	1	2	2	2	−0.0392	−0.0003	0.0242	−0.0087	−0.1847	0.0199	−0.0003	−0.1866
9	1	1	1	2	1	1	1	0.0758	0.0002	0.0464	0.0212	0.0103	−0.0517	0.0008	0.3002
10	1	1	1	2	1	1	2	0.1390	0.0005	0.0847	0.0410	−0.0061	−0.0735	−0.0001	0.5629
11	1	1	1	2	1	2	1	0.0969	0.0003	0.0625	0.0268	0.0127	−0.0156	−0.0001	0.3842
12	1	1	1	2	1	2	2	0.1234	0.0003	0.0993	0.0355	0.0199	−0.0232	0.0002	0.4851
13	1	1	1	2	2	1	1	0.0485	0.0001	0.0444	0.0143	0.1755	−0.0609	0.0022	0.1925
14	1	1	1	2	2	1	2	−0.0167	−0.0001	0.0385	0.0036	−0.1037	−0.0748	0.0022	−0.0702
15	1	1	1	2	2	2	1	0.0599	0.0001	0.0512	0.0165	0.2165	−0.0188	−0.0004	0.2383
16	1	1	1	2	2	2	2	0.0925	0.0002	0.1150	0.0299	0.3389	−0.0243	−0.0004	0.3663
17	1	1	2	1	1	1	1	0.0356	0.0000	0.0445	0.0046	0.0069	−0.0470	0.0034	0.1371
18	1	1	2	1	1	1	2	−0.1395	0.0000	−0.1964	−0.0165	0.0091	−0.0162	0.0030	−0.5584
19	1	1	2	1	1	2	1	0.0943	0.0000	0.1238	0.0119	0.0108	−0.0154	−0.0009	0.3697
20	1	1	2	1	1	2	2	0.0331	0.0000	0.0364	0.0051	0.0176	0.0021	−0.0004	0.1241
21	1	1	2	1	2	1	1	0.0319	0.0000	0.0413	0.0044	0.1179	−0.0486	0.0017	0.1256
22	1	1	2	1	2	1	2	0.0328	0.0000	0.0488	0.0030	0.1549	−0.0815	0.0007	0.1215
23	1	1	2	1	2	2	1	0.0518	0.0000	0.0663	0.0071	0.1829	−0.0169	−0.0002	0.2040
24	1	1	2	1	2	2	2	0.0756	−0.0001	0.1045	0.0109	0.2996	−0.0263	0.0000	0.3059
25	1	1	2	2	1	1	1	0.0457	0.0001	0.0287	0.0124	0.0093	−0.0348	0.0009	0.1818
26	1	1	2	2	1	1	2	0.0710	0.0003	0.0375	0.0223	0.0070	−0.0543	0.0002	0.2923
27	1	1	2	2	1	2	1	0.0643	0.0002	0.0421	0.0167	0.0107	−0.0102	−0.0002	0.2530
28	1	1	2	2	1	2	2	0.0954	0.0003	0.0580	0.0252	0.0238	−0.0159	0.0000	0.3701
29	1	1	2	2	2	1	1	0.0434	0.0001	0.0273	0.0112	0.1576	−0.0521	0.0013	0.1678
30	1	1	2	2	2	1	2	0.0319	0.0001	0.0246	0.0101	0.1182	−0.0929	0.0008	0.1349
31	1	1	2	2	2	2	1	0.0517	0.0001	0.0324	0.0128	0.1825	−0.0162	−0.0002	0.1968
32	1	1	2	2	2	2	2	0.1106	0.0003	0.0705	0.0285	0.4053	−0.0321	−0.0003	0.4257
33	1	2	1	1	1	1	1	0.0016	0.0000	0.0025	0.0004	−0.0020	0.0006	−0.0002	0.0054
34	1	2	1	1	1	1	2	−0.0044	0.0000	−0.0006	−0.0003	−0.0142	0.0099	0.0000	−0.0153
35	1	2	1	1	1	2	1	0.0031	0.0000	0.0045	0.0006	−0.0007	−0.0005	0.0001	0.0102
36	1	2	1	1	1	2	2	−0.0133	−0.0001	0.0051	−0.0025	−0.0096	0.0012	0.0000	−0.0576
37	1	2	1	1	2	1	1	−0.0079	0.0000	0.0025	−0.0015	−0.0341	0.0061	0.0005	−0.0337
38	1	2	1	1	2	1	2	−0.0605	−0.0002	0.0020	−0.0120	−0.2408	0.0418	0.0011	−0.2408
39	1	2	1	1	2	2	1	−0.0021	0.0000	0.0035	−0.0004	−0.0122	0.0015	−0.0001	−0.0116
40	1	2	1	1	2	2	2	−0.0404	−0.0002	0.0060	−0.0078	−0.1632	0.0157	−0.0001	−0.1660
41	1	2	1	2	1	1	1	0.0389	0.0001	0.0241	0.0109	0.0046	−0.0260	0.0003	0.1544
42	1	2	1	2	1	1	2	0.0759	0.0002	0.0465	0.0218	−0.0049	−0.0357	−0.0001	0.3033
43	1	2	1	2	1	2	1	0.0485	0.0001	0.0317	0.0137	0.0057	−0.0079	0.0000	0.1939
44	1	2	1	2	1	2	2	0.0562	0.0002	0.0492	0.0172	0.0075	−0.0113	0.0001	0.2256
45	1	2	1	2	2	1	1	0.0212	0.0001	0.0218	0.0068	0.0775	−0.0273	0.0011	0.0864
46	1	2	1	2	2	1	2	−0.0153	−0.0001	0.0222	0.0003	−0.0841	−0.0278	0.0012	−0.0660
47	1	2	1	2	2	2	1	0.0261	0.0001	0.0243	0.0079	0.0969	−0.0084	−0.0002	0.1080
48	1	2	1	2	2	2	2	0.0336	0.0001	0.0558	0.0132	0.1272	−0.0086	−0.0002	0.1400
49	1	2	2	1	1	1	1	0.0106	0.0000	0.0121	0.0014	0.0013	−0.0120	0.0008	0.0395
50	1	2	2	1	1	1	2	−0.0275	0.0000	−0.0422	−0.0031	0.0030	−0.0048	0.0007	−0.1140
51	1	2	2	1	1	2	1	0.0241	0.0000	0.0304	0.0031	0.0020	−0.0039	−0.0002	0.0933
52	1	2	2	1	1	2	2	0.0065	0.0000	0.0080	0.0013	0.0025	0.0006	−0.0001	0.0261
53	1	2	2	1	2	1	1	0.0058	0.0000	0.0087	0.0010	0.0224	−0.0092	0.0003	0.0247
54	1	2	2	1	2	1	2	0.0133	0.0000	0.0171	0.0011	0.0503	−0.0151	0.0000	0.0434
55	1	2	2	1	2	2	1	0.0092	0.0000	0.0135	0.0015	0.0341	−0.0032	0.0000	0.0395
56	1	2	2	1	2	2	2	0.0085	0.0000	0.0188	0.0018	0.0428	−0.0043	0.0000	0.0442
57	1	2	2	2	1	1	1	0.0233	0.0001	0.0143	0.0064	0.0044	−0.0172	0.0004	0.0928
58	1	2	2	2	1	1	2	0.0421	0.0001	0.0241	0.0123	0.0038	−0.0277	0.0000	0.1699
59	1	2	2	2	1	2	1	0.0311	0.0001	0.0196	0.0084	0.0049	−0.0050	−0.0001	0.1235
60	1	2	2	2	1	2	2	0.0459	0.0001	0.0283	0.0126	0.0112	−0.0080	0.0000	0.1813
61	1	2	2	2	2	1	1	0.0203	0.0001	0.0128	0.0055	0.0750	−0.0242	0.0006	0.0802
62	1	2	2	2	2	1	2	0.0183	0.0001	0.0135	0.0055	0.0646	−0.0441	0.0003	0.0743
63	1	2	2	2	2	2	1	0.0231	0.0001	0.0144	0.0061	0.0834	−0.0074	−0.0001	0.0904
64	1	2	2	2	2	2	2	0.0507	0.0001	0.0326	0.0139	0.1899	−0.0152	−0.0001	0.1999
65	2	1	1	1	1	1	1	0.0378	0.0000	0.0207	0.0048	0.0290	−0.0380	0.0029	0.1113
66	2	1	1	1	1	1	2	−0.2351	0.0001	−0.0975	−0.0361	−0.0329	−0.0576	0.0026	−0.6919
67	2	1	1	1	1	2	1	0.1343	0.0000	0.0647	0.0189	0.0499	−0.0152	−0.0007	0.3954
68	2	1	1	1	1	2	2	0.3467	−0.0001	0.1547	0.0512	0.0788	−0.0389	−0.0005	1.0180
69	2	1	1	1	2	1	1	0.1698	−0.0001	0.0748	0.0254	0.4938	−0.2096	0.0086	0.4986
70	2	1	1	1	2	1	2	−0.2011	0.0002	−0.0747	−0.0327	−0.5586	−0.2514	0.0084	−0.5974
71	2	1	1	1	2	2	1	0.2952	−0.0001	0.1297	0.0442	0.8475	−0.0721	−0.0025	0.8678
72	2	1	1	1	2	2	2	0.4756	−0.0003	0.2009	0.0729	1.3393	−0.0855	−0.0023	1.3946
73	2	1	1	2	1	1	1	0.0499	0.0001	0.0121	0.0093	0.0043	−0.0293	0.0020	0.1514
74	2	1	1	2	1	1	2	−0.1036	0.0003	−0.1106	−0.0052	0.0106	−0.0239	0.0018	−0.2958
75	2	1	1	2	1	2	1	0.0622	−0.0001	0.0573	0.0060	0.0040	−0.0077	−0.0006	0.1886
76	2	1	1	2	1	2	2	0.1446	−0.0004	0.1641	0.0093	0.0101	−0.0089	−0.0007	0.4325
77	2	1	1	2	2	1	1	0.0271	−0.0003	0.0848	−0.0051	0.0739	−0.0234	0.0007	0.0826
78	2	1	1	2	2	1	2	0.0553	0.0006	−0.1133	0.0271	0.1795	−0.0522	0.0001	0.1615
79	2	1	1	2	2	2	1	0.0253	−0.0005	0.1463	−0.0136	0.0672	−0.0059	0.0000	0.0781
80	2	1	1	2	2	2	2	0.0648	−0.0009	0.2521	−0.0186	0.1714	−0.0186	0.0002	0.1962
81	2	1	2	1	1	1	1	0.0852	0.0002	0.0602	0.0083	0.0049	−0.0496	0.0013	0.2524
82	2	1	2	1	1	1	2	−0.0012	0.0005	0.0398	−0.0096	0.0273	−0.0594	0.0013	−0.0123
83	2	1	2	1	1	2	1	0.1288	0.0002	0.0769	0.0158	0.0080	−0.0176	−0.0003	0.3841
84	2	1	2	1	1	2	2	0.1339	−0.0002	0.0365	0.0245	−0.0100	−0.0130	−0.0003	0.3954
85	2	1	2	1	2	1	1	0.0324	−0.0002	−0.0077	0.0095	0.0825	−0.0332	−0.0006	0.0971
86	2	1	2	1	2	1	2	0.1564	0.0009	0.1627	0.0045	0.4638	−0.0187	−0.0018	0.4615
87	2	1	2	1	2	2	1	0.0527	−0.0004	−0.0163	0.0162	0.1360	−0.0126	0.0001	0.1582
88	2	1	2	1	2	2	2	−0.0511	−0.0012	−0.1324	0.0153	−0.1694	0.0117	0.0003	−0.1455
89	2	1	2	2	1	1	1	0.0194	0.0000	0.0096	0.0030	0.0165	−0.0207	0.0016	0.0598
90	2	1	2	2	1	1	2	−0.1286	0.0001	−0.0576	−0.0182	−0.0184	−0.0323	0.0014	−0.3734
91	2	1	2	2	1	2	1	0.0714	0.0000	0.0348	0.0105	0.0278	−0.0081	−0.0004	0.2138
92	2	1	2	2	1	2	2	0.1929	0.0000	0.0904	0.0285	0.0463	−0.0218	−0.0003	0.5727
93	2	1	2	2	2	1	1	0.0954	0.0000	0.0454	0.0140	0.2804	−0.1176	0.0049	0.2829
94	2	1	2	2	2	1	2	−0.1143	0.0001	−0.0529	−0.0164	−0.3128	−0.1466	0.0048	−0.3336
95	2	1	2	2	2	2	1	0.1635	0.0000	0.0782	0.0238	0.4725	−0.0402	−0.0014	0.4835
96	2	1	2	2	2	2	2	0.2760	−0.0001	0.1296	0.0409	0.7867	−0.0511	−0.0013	0.8177
97	2	2	1	1	1	1	1	0.0151	0.0000	0.0093	0.0017	0.0051	−0.0112	0.0006	0.0444
98	2	2	1	1	1	1	2	−0.0394	0.0001	−0.0126	−0.0068	−0.0031	−0.0150	0.0006	−0.1160
99	2	2	1	1	1	2	1	0.0349	0.0000	0.0183	0.0046	0.0088	−0.0042	−0.0001	0.1027
100	2	2	1	1	1	2	2	0.0708	0.0000	0.0312	0.0107	0.0114	−0.0077	−0.0001	0.2072
101	2	2	1	1	2	1	1	0.0306	0.0000	0.0125	0.0049	0.0870	−0.0370	0.0014	0.0893
102	2	2	1	1	2	1	2	−0.0192	0.0001	0.0018	−0.0050	−0.0534	−0.0410	0.0012	−0.0601
103	2	2	1	1	2	2	1	0.0530	−0.0001	0.0215	0.0085	0.1501	−0.0128	−0.0004	0.1556
104	2	2	1	1	2	2	2	0.0708	−0.0002	0.0231	0.0126	0.1941	−0.0121	−0.0003	0.2056
105	2	2	1	2	1	1	1	0.0182	0.0000	0.0038	0.0036	−0.0046	−0.0069	0.0003	0.0559
106	2	2	1	2	1	1	2	0.0093	0.0001	−0.0237	0.0058	0.0133	−0.0003	0.0003	0.0321
107	2	2	1	2	1	2	1	0.0034	0.0000	0.0146	−0.0008	−0.0094	−0.0008	−0.0001	0.0130
108	2	2	1	2	1	2	2	−0.0092	−0.0002	0.0396	−0.0065	−0.0146	0.0044	−0.0002	−0.0237
109	2	2	1	2	2	1	1	−0.0259	−0.0001	0.0202	−0.0077	−0.0784	0.0367	−0.0017	−0.0749
110	2	2	1	2	2	1	2	0.0777	0.0002	−0.0246	0.0196	0.2262	0.0355	−0.0020	0.2273
111	2	2	1	2	2	2	1	−0.0547	−0.0002	0.0344	−0.0155	−0.1604	0.0135	0.0006	−0.1591
112	2	2	1	2	2	2	2	−0.0856	−0.0004	0.0593	−0.0247	−0.2490	0.0127	0.0006	−0.2495
113	2	2	2	1	1	1	1	0.0477	0.0001	0.0345	0.0044	−0.0047	−0.0228	0.0001	0.1408
114	2	2	2	1	1	1	2	0.0641	0.0003	0.0566	0.0035	0.0269	−0.0246	0.0002	0.1866
115	2	2	2	1	1	2	1	0.0502	0.0001	0.0328	0.0055	−0.0082	−0.0076	0.0000	0.1489
116	2	2	2	1	1	2	2	−0.0009	−0.0002	−0.0150	0.0029	−0.0285	0.0015	−0.0001	−0.0022
117	2	2	2	1	2	1	1	−0.0242	−0.0001	−0.0250	−0.0006	−0.0792	0.0347	−0.0028	−0.0706
118	2	2	2	1	2	1	2	0.1589	0.0006	0.1279	0.0119	0.4581	0.0572	−0.0035	0.4675
119	2	2	2	1	2	2	1	−0.0443	−0.0003	−0.0453	−0.0012	−0.1388	0.0112	0.0007	−0.1296
120	2	2	2	1	2	2	2	−0.1645	−0.0007	−0.1428	−0.0099	−0.4842	0.0317	0.0008	−0.4826
121	2	2	2	2	1	1	1	0.0071	0.0000	0.0038	0.0011	0.0033	−0.0060	0.0004	0.0224
122	2	2	2	2	1	1	2	−0.0232	0.0000	−0.0099	−0.0032	−0.0024	−0.0086	0.0003	−0.0655
123	2	2	2	2	1	2	1	0.0180	0.0000	0.0095	0.0027	0.0055	−0.0022	−0.0001	0.0548
124	2	2	2	2	1	2	2	0.0421	0.0000	0.0207	0.0064	0.0087	−0.0047	−0.0001	0.1264
125	2	2	2	2	2	1	1	0.0192	0.0000	0.0097	0.0029	0.0566	−0.0233	0.0009	0.0577
126	2	2	2	2	2	1	2	−0.0155	0.0001	−0.0069	−0.0023	−0.0406	−0.0293	0.0008	−0.0443
127	2	2	2	2	2	2	1	0.0321	0.0000	0.0163	0.0047	0.0929	−0.0079	−0.0002	0.0958
128	2	2	2	2	2	2	2	0.0516	−0.0001	0.0250	0.0080	0.1477	−0.0098	−0.0002	0.1546
